# Code dependence of calculated crystalline electron densities. Possible lessons for quantum crystallography

**DOI:** 10.1107/S2052252525001721

**Published:** 2025-03-28

**Authors:** Bruno Landeros-Rivera, Julia Contreras-García, Ángel Martín Pendás

**Affiliations:** a Departmento de Química Inorgánica y Nuclear, Universidad Nacional Autónoma De México, 04510Ciudad de México, México; b Laboratoire de Chimie Théorique, Sorbonne Universite and CNRS, 4 Pl. Jussieu, F. 75005Paris, France; c Departmento Química Física y Analítica, Universidad de Oviedo, 33006Oviedo, Spain; Warsaw University, Poland

**Keywords:** computational modeling, density functional theory, charge, spin and momentum densities, properties of solids, quantum crystallography, *VASP*, *Quantum Espresso*

## Abstract

The use of electronic structure methods in crystallographic data analysis, the now well known field of quantum crystallography, aids in the solution of several problems in X-ray diffraction refinement, as well as opening new avenues to access a whole new set of experimentally available observables. A key ingredient in quantum crystallography is the theoretically derived electron density, ρ, obtained from standard electronic structure codes. Here, we introduce a factor that has not been carefully considered until now. As we demonstrate, theoretically derived ρ values depend not only on the set of computational conditions used to obtain them but also on the particular computational code selected for this task. We recommend that all quantum crystallographers carefully check the convergence of ρ before undertaking any serious study.

## Abstract 2

The development of quantum crystallography depends on the availability of reliable theoretical electron densities. This work demonstrates a non-negligible code dependence of these densities and warns against their blind use.

## 1. Introduction

Quantum crystallography (Grabowsky *et al.*, 2017 ▸; Genoni & Macchi, 2020 ▸; Macchi, 2022 ▸; Krawczuk & Genoni, 2024 ▸) (QC) is revolutionizing how we understand and use X-ray diffraction (XRD) by integrating advanced quantum chemical calculations into the standard techniques used in crystallographic data analysis. The field has its origins in the early days of quantum physics, when scientists like Weiss and DeMarco (Weiss & De Marco, 1958 ▸; Weiss & De Marco, 1959 ▸; Weiss & De Marco, 1965 ▸) sought to use XRD to derive electron configurations for atoms in crystals. Significant progress in this area, however, only occurred later in the mid-20th century, when several proposals culminated in the Clinton–Massa equations (Clinton *et al.*, 1969 ▸; Clinton & Massa, 1972 ▸). After several relevant contributions (Huang *et al.*, 1999 ▸; Massa & Matta, 2018 ▸; Henderson, 2018 ▸; Genoni *et al.*, 2018 ▸), in 1998 Jayatilaka introduced the X-ray restrained wavefunction (XRW) method (Jayatilaka, 1998 ▸) that underlies most of the recent advances in the field. Grossly speaking, in XRW we seek to variationally optimize the energy coming from an externally parametrized wavefunction while simultaneously maximizing its agreement with experimentally determined structure factors. The introduction of the XRW method represented a major leap forward, since it provided direct access to some properties (*e.g.* energies, polarizabilities) which could otherwise be calculated only approximately in methods such as in the Hansen–Coppens multipole refinement (Hansen & Coppens, 1978 ▸), in which only a first-order density matrix is available. After several extensions, QC techniques are now used to cope with problems that range from relativistic or correlation effects in experimentally determined electron densities (Genoni *et al.*, 2017 ▸; Hupf *et al.*, 2023 ▸; Bučinský *et al.*, 2016 ▸; Bučinský *et al.*, 2019 ▸; Podhorský *et al.*, 2021 ▸) to experimentally driven chemical bonding analyses (Ernst *et al.*, 2020 ▸), having reached a considerable level of maturity. Furthermore, QC is not just limited to XRD but also incorporates other experimental techniques such as electron diffraction (Gruene *et al.*, 2021 ▸) and neutron scattering (Hoser & Madsen, 2016 ▸; Hoser & Madsen, 2017 ▸). When combined with quantum mechanical models, these methods provide a comprehensive understanding of many properties of materials. The integration of quantum chemistry into crystallography has also led to the development of new techniques for refining crystal structures, such as Hirshfeld atom refinement (HAR) (Capelli *et al.*, 2014 ▸), which uses quantum-mechanically derived scattering factors to improve the accuracy of atomic positions and, indirectly, of thermal parameters in crystal structures. HAR has convincingly shown, for instance, that in some cases neutron diffraction is no longer needed to refine accurate hydrogen positions if high-quality XRD experiments are available (Woińska *et al.*, 2014 ▸; Woińska *et al.*, 2016 ▸). Notwithstanding this, although the anisotropic displacement parameters (ADPs) of hydrogen atoms are commonly expected to be larger than those of heavier atoms, non-positive definite values can be obtained from HAR despite measuring data at low temperatures, high resolution, large redundancy and using high levels of theory in the self-consistent field calculations (Chocolatl Torres *et al.*, 2021 ▸; Novelli *et al.*, 2021 ▸). For instance, the ADP of the hydrogen atom of a strong intramolecular hydrogen bond found in the 8-hydroxyquinolinium hydrogen maleate crystal becomes non-positive definite despite the fact that the XRD data were collected at 15 K, with a redundancy of 5.12; a resolution of 0.43 Å; and the employment of GGA, hybrid and meta-GGA functionals with triple-*Z* basis sets (Malaspina *et al.*, 2021 ▸). Thus, in these cases the enhanced rigid-bond restraints employed in conventional crystallography are required. Moreover, the refined *X*—H bond lengths are also affected by the quantum mechanical model [Hamiltonian, basis set, crystal packing representations *etc.* (Landeros-Rivera *et al.*, 2023 ▸)]. Thus, in the context of general QC, the accuracy of the experimentally derived electron densities becomes critical, as any inaccuracies will propagate through the analysis (Landeros-Rivera *et al.*, 2021 ▸), leading to flawed predictions about the reactivity, stability or properties of the materials under study. This is particularly problematic, for instance, when analyzing weak interactions, charge transfer processes or bond formation (Saunders *et al.*, 2021 ▸).

After years of steady development, the joint use of high-resolution structure factors together with XRW seems to have reached such a level of accuracy in the determination of ‘experimental’ electron densities that a debate in the community has been sparked about the ability of QC techniques to compete or even outperform theoretically obtained densities. However, the current limitations of the XRW approach, particularly its inability to fully account for crystal effects, suggest the need to develop a fully periodic version of the method before such questions can be fully answered (Genoni & Martín Pendás, 2024 ▸). In the meantime, several lines of reasoning are being actively explored.

On the one hand, the XRW approach is starting to be explored as a way to improve the accuracy of density functional approximations (DFAs) in density functional theory (DFT) calculations. Some studies, such as that by Medvedev *et al.* (2017 ▸), have shown that many modern DFAs prioritize energy accuracy at the expense of electron density accuracy. In fact, the empirical fitting of new functionals is usually done against energetic data without considering the quality of the electron densities they produce at any of its stages. As a result, many modern DFAs deviate significantly from the exact electron density as provided, for example, by gold-standard coupled-cluster calculations (Medvedev *et al.*, 2017 ▸), even if they provide accurate energies for specific systems. The XRW method offers a potential solution to this problem by providing access to more reliable electron densities that can be used to improve DFAs. In a recent study (Genoni & Martín Pendás, 2024 ▸), it has been shown that final XRW densities are indeed fairly insensitive to the functional used in the restrained optimization of the wavefunction, converging towards a so-called ‘consensus’ density that differs substantially from any of those provided by any of the tested functionals. To what extent this deviation results from the failure of the DFAs or the flaws of the molecular implementation of XRW remains to be known.

Here we explore another important issue that needs to be properly settled while we await a periodic implementation of the XRW methodology. Though this has already been implemented in HAR (Ruth *et al.*, 2022 ▸), its development in XRW involves more complications that are outside the scope of this work. Whether at one point in time or another, a comparison between XRW and theoretical densities will be needed. Much as in the DFA case, decades of sustained development have led to a set of standardized, reliable solid-state computational codes that can solve the electronic structure of periodic solids. In almost every case they use DFT to provide a single-determinant wavefunction built from one-electron crystal orbitals. The latter are typically linear combinations of atom-centered basis functions [like in the *CRYSTAL* code (Erba *et al.*, 2023 ▸)] or, more commonly, plane waves, that avoid the high-frequency oscillations present close to the nuclei through the use of pseudopotentials (PPs) (Kresse & Hafner, 1993 ▸; Kresse & Hafner, 1994 ▸; Kresse & Furthmüller, 1996*a* ▸; Kresse & Furthmüller, 1996*b* ▸; Kresse & Joubert, 1999 ▸; Giannozzi *et al.*, 2009 ▸; Giannozzi *et al.*, 2017 ▸). Other less frequent possibilities (*i.e.* truncated or numerical basis sets) also exist.

How do the plethora of specific approximations, implemented algorithms *etc.* influence the computed electron densities from different codes in diverse crystals? To pinpoint this possible code-dependency problem as much as possible, we will restrict ourselves to the *VASP* and *Quantum Espresso* (*QE*) codes (Kresse & Hafner, 1993 ▸; Kresse & Hafner, 1994 ▸; Kresse & Furthmüller, 1996*a* ▸; Kresse & Furthmüller, 1996*b* ▸; Kresse & Joubert, 1999 ▸; Giannozzi *et al.*, 2009 ▸; Giannozzi *et al.*, 2017 ▸), which should provide exactly the same answer under the same computational conditions. We will also demonstrate that this is not always the case, so that this source of uncertainty will need to be taken into account in the future.

We have selected a collection of crystals that span a wide range of chemical bonding situations. We start with NaCl and urea. The first is a clearly ionic system in which no major chemical bonding density redistributions occur, so we should not expect significant differences in results from different codes or computational conditions. Urea is one of the favorite systems used in crystallographic benchmarks. Then we shift to SbH_4_, a hydrogen-rich system that shows superconducting behavior whose density is difficult to model due to the diffuse character of the hydrogen atoms. As we will show, the code dependence leads to non-negligible differences in the estimation of properties here. Finally, we deal with magnesium bis(hydrogen maleate) hexahydrate (Mg[C_4_H_3_O_4_]_2_·6H_2_O), MgM in the following, a system that shows covalent, ionic and dative interactions, as well as several intermolecular hydrogen bonds, together with a strong resonance-assisted intramolecular [O⋯H⋯O]^−^ hydrogen bond (RAHB) within the maleate anion (Malaspina *et al.*, 2020 ▸). Moreover, all the atomic coordinates, including those of the hydrogen atoms, were accurately determined from neutron-diffraction data collected at 12 K with a very good resolution (0.6 Å) (Malaspina *et al.*, 2017 ▸). Since our goal is to examine code-dependent results, we have selected both LDA and GGA DFAs in our calculations to exclude any explicit functional dependence.

### 1.1. Computational details

Provided our goal is to uncover to what extent the use of different computational codes impact computed electron densities, some care must be taken when choosing a set of input parameters. Although a perfect match between the *VASP* and *QE* codes is close to impossible, for instance the standard projector augmented wave (PAW) pseudopotentials used in the former are different from those used in the latter, many of the tunable knobs in both codes can be put in a one-to-one correspondence. This way we expect to attain as comparable as possible solutions. In keeping with recommendations from the developers of both codes, we have selected:

Energy cutoff: 600 eV. This parameter controls how many plane waves are included in the expansion of the wavefunction, *i.e.* only those plane waves whose kinetic energy is lower than the energy cutoff are used for the Fourier expansion. This value was selected based on the *QE* pseudopotential (see below), using the *Materials Cloud* platform recommendation (Talirz *et al.*, 2020 ▸). This parameter is controlled by the ‘ENCUT’ and ‘ecutwfc’ keywords in *VASP* and *QE*, respectively.

Energy threshold: 10^−6^ eV. This is the convergence criterion for the energy-minimization process. It is controlled by the ‘EDIFF’ and ‘conv_thr’ keywords in *VASP* and *QE*, respectively.

Diagonalization algorithm: Davidson/RMM-DIIS. This method is employed for matrix diagonalization. It is selected by the ‘ALGO = Fast’ and ‘diagonalization = rmm-davidson’ keywords in *VASP* and *QE*, respectively.

FFT mesh: 2**G_cut_**. This is the fast Fourier transform (FFT) grid used for the conversion between real and reciprocal spaces. It contains all the wavevectors up to 2**G_cut_**, where 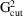 is equal to the energy cutoff (see above) multiplied by 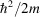. This value was selected with the ‘PREC = Accurate’ option in *VASP*. Then, the number of grid points in the first, second and third lattice vectors found in the *VASP* output (NGX, NGY and NGZ, respectively) were used as input values for the FFT grid in *QE* (using the ‘nr1s’, ‘nr2s’ and ‘nr3s’ keywords, respectively).

Fine FFT mesh: 4**G_cut_**. This is the finer FFT grid where local potentials are evaluated. This value was also controlled with the ‘PREC = Accurate’ option in *VASP*. The number of grid points in the first, second and third lattice vectors are twice those of the first FFT mesh. In *QE* this parameter is controlled by the ‘nr1’, ‘nr2’ and ‘nr3’ keywords, respectively. It is known that this parameter has a considerable impact on the accuracy of the calculated electron densities, see below.

K-POINTS: Monkhorst–Pack grids. This is the number of points used for sampling the Brillouin zone. This grid depends on the unit cell of each system, although the same value was selected for each one in *VASP* and *QE*. In both codes, the automatic generation algorithm was employed.

Pseudopotentials: *VASP* LDA and PBE standard PAW potentials. On the one hand, as mentioned before, these are employed to avoid high-frequency oscillations close the nuclei. On the other hand, they speed up the calculations by allowing only the valence electrons to be considered in the calculations. The *VASP* pseudopotentials are provided with the code. Contrarily, the LDA, PBE and PW91 PAW pseudopotentials used in *QE* were computed through the *ld1.x QE* program with the help of the *PSLibrary* by Dal Corso (2014 ▸). Table S1 of the supporting information contains the crystallographic data and computational details for each of the selected systems. The most relevant atom labels and bond distances are depicted in Fig. S1 of the supporting information.

The same fixed unit-cell parameters and atomic coordinates were used in both program energy-minimization processes, which were taken from the sources mentioned in the *Introduction*[Sec sec1]. No smearing (partial occupancy of different states) was used in any of the computations. The single-point calculations were carried out with the LDA-PZ, PW91 and PBE GGA density functionals. The topological analysis of the electron density was performed with the *Critic2* program (Otero-de-la Roza *et al.*, 2014 ▸).

## 2. The NaCl crystal

**Figure 1:**
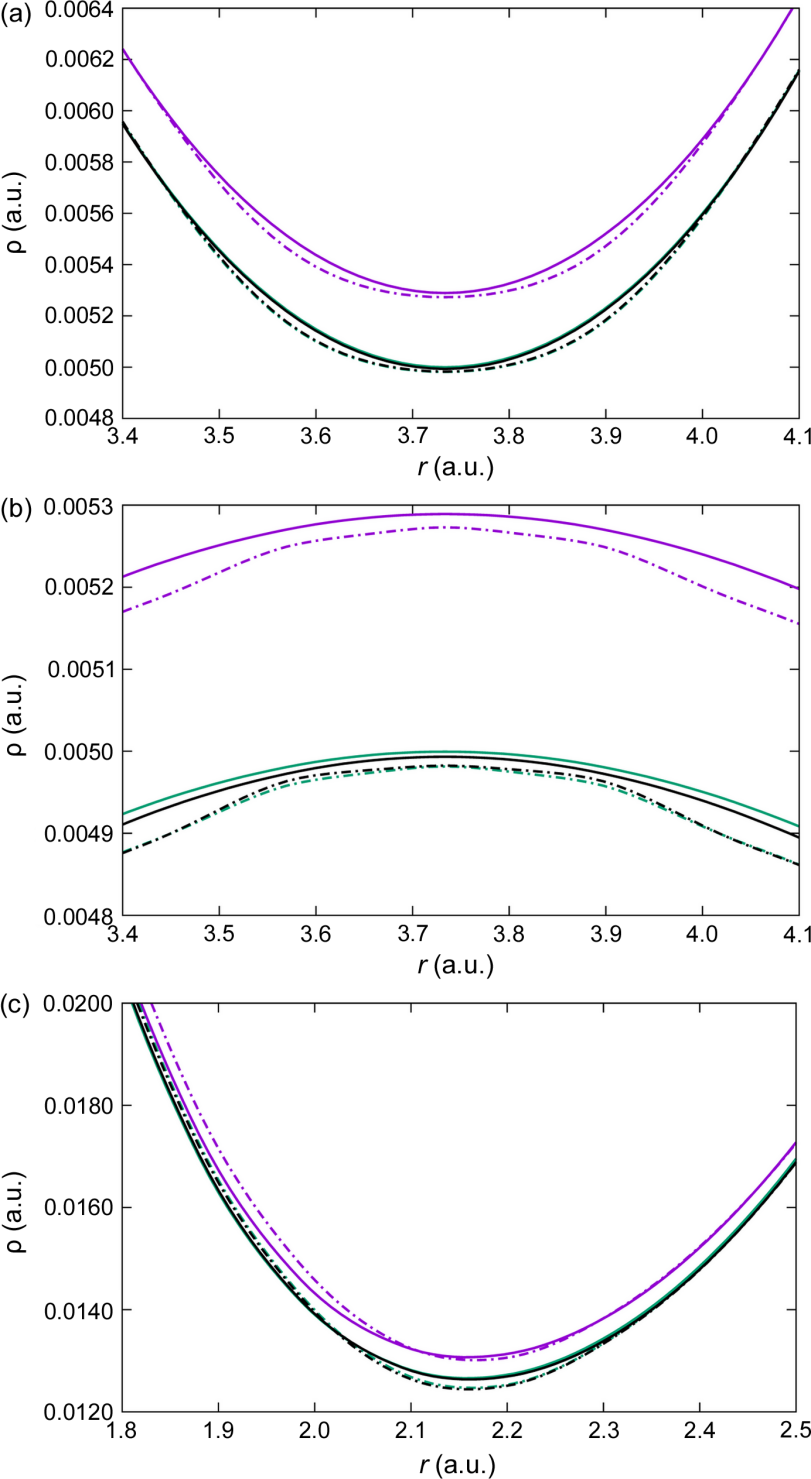
Electron densities as calculated with *VASP* (full lines) and *QE* (dashed lines) along the Cl—Cl (top), Na—Na (middle) and Na—Cl (bottom) internuclear axes in the NaCl crystal. Different DFAs are color coded: LDA in purple, PBE in green and PW91 in black. Distances are measured from the left nucleus of each pair. The right nucleus of the pair is located at the first- (Na—Cl) or second-neighbor distance (Na—Na, Cl—Cl), at 5.28 and 7.47 a.u., respectively. Notice that several PBE and PW91 curves, particularly along the Na—Cl line, mostly overlap.

We start with the simple NaCl crystal, computed in its B1 (f.c.c.) phase with the lattice parameter *a* = 5.58813 Å. We expect that, owing to its large ionicity, no major discrepancies between the codes will emerge. Since in QC we are mainly interested in chemically relevant problems, we will not consider the nuclear cores (where large deviations are expected and indeed occur) but will concentrate on bonding regions instead. Fig. 1 ▸ shows the electron density along the Cl—Cl, Na—Na and Na—Cl lines. It is well known that Na—Cl and Cl—Cl bond critical points (BCPs) exist in this system (Martín Pendás *et al.*, 1997 ▸), while the Na—Na midpoint is a ring critical point, so we observe minima and maxima, respectively.

Our expectations are met this time. Overall, differences are larger among different DFAs than between the two codes for a chosen DFA. The LDA provides consistently larger densities along the Cl—Cl and Na—Cl lines (with BCPs) than any of the PBE or PW91 DFAs, which are almost degenerate with each other. The contrary is true along the Na—Na direction. This is the expected behavior from what is known about the delocalization error in DFT (Cohen *et al.*, 2012 ▸; Mori-Sánchez *et al.*, 2008 ▸), with gradient-corrected (GGA) functionals localizing more than pure local DFAs. Provided that the number of electrons is conserved, we expect an electron flow from bonding to non-bonding regions as we shift from LDA to GGA, as can be clearly seen in Fig. 1 ▸. That said, there are several interesting remarks. Firstly, a change in the code changes the position of the Na—Cl BCP (not fixed by symmetry, as happens along the Cl—Cl direction). Albeit slight, the change is significant. Taking LDA as an example, the bonded radii of Na are 1.951 and 1.946 a.u. in *VASP* and *QE*, respectively. The curvatures are obviously even more sensitive. The Laplacian of the density, 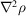, changes non-negligibly from 0.0578 to 0.0818 a.u. in the same order. Beyond a shift in the density values the GGA results are similar, with bonded Na radii along the Na—Cl direction equal to 1.948 and 1.945 a.u. and 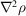 = 0.0578 and 0.0876 a.u., respectively. In this regard, the two codes provide consistent, yet slightly different results across different DFAs. Interestingly, if ρ is stiffer (larger 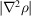) at the NaCl BCP for the *QE* code, the contrary is true at the Cl—Cl counterpart, with *VASP* and *QE* Laplacians equal to 0.0125 and 0.002 a.u., respectively. We stress that relatively small local density differences may lead to substantial differences at the global level. As a rule of thumb, pertubation theory can be used to show a charge transfer of about 1 m*e* (millielectron) is typically associated with an equivalent energy change in atomic units, with 1 m*E*_h_ ≃ 0.6 kcal mol^−1^ (the origin of this rule of thumb lies in noticing that the potential energy changes in such a transfer by 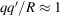
 m*E*_h_ and then invoking virial arguments to transform potential into total energies). This value can be considered a large amount in many cases, such as when trying to predict or rationalize magnetic phases, for example. Finally, the middle panel of Fig. 1 ▸ shows some spurious wiggles in the *QE* data not present in their *VASP* counterparts. As we will show shortly, these are due to an insufficiently large fine-FFT grid. We thus recommend using large fine-FFT meshes to avoid these artifacts.

**Figure 2:**
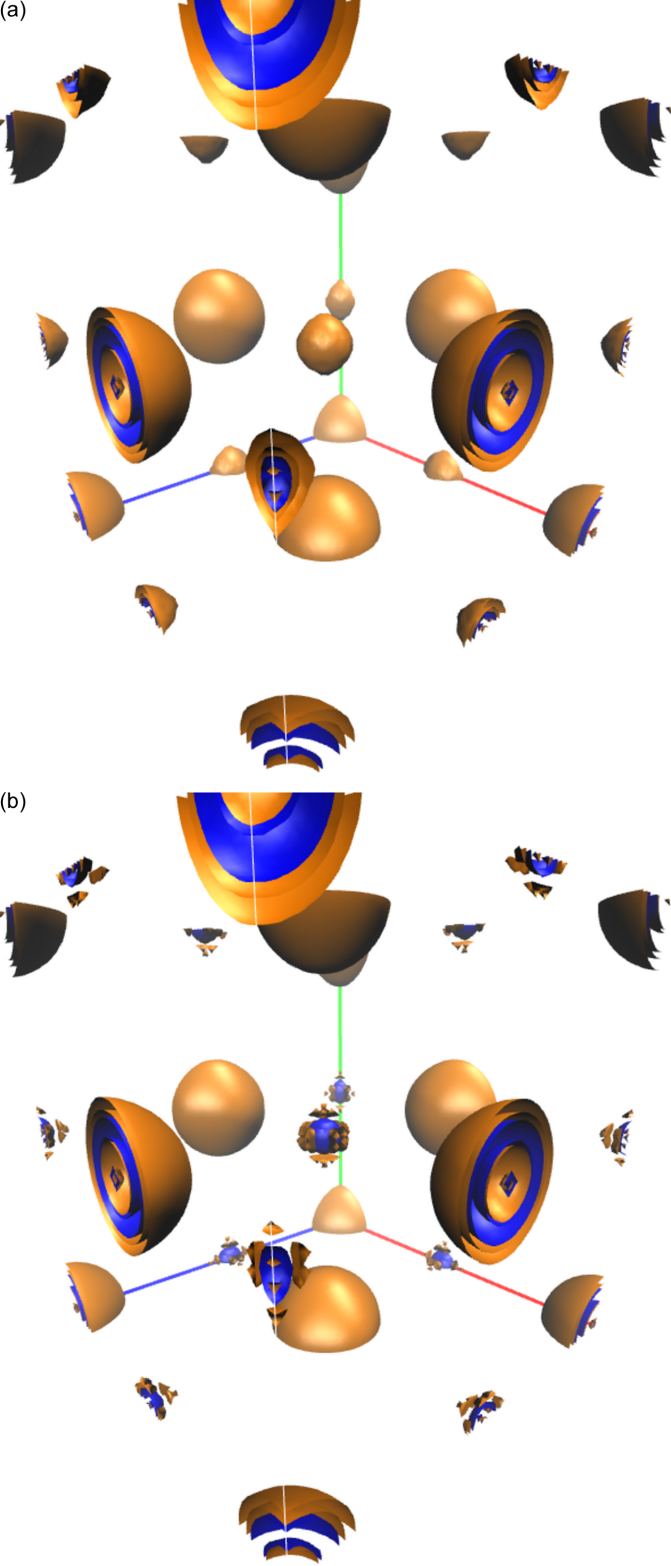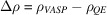 difference density maps for NaCl as obtained with the LDA (top panel) and PW91 (bottom panel) DFAs with 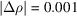 a.u. Positive and negative isosurfaces are depicted in blue and orange, respectively. The Cl and Na moieties can be isolated as the large and small spheres, respectively.

Having explored the density differences along the most relevant directions in the NaCl crystal, we turn to the overall code differences within the unit cell. Fig. 2 ▸ shows quasi-spherical oscillating patterns for the Cl^−^ anion that survive the change in DFA, pointing towards a well defined pattern in the *VASP*/*QE* differences. The behavior of the Na^+^ cation seems to be slightly more dependent on the DFA, although the density differences in its surroundings are smaller, as expected from its smaller role in binding.

Although our aim here is simply to unveil the variability of computed densities emerging from the computer code in use and not to examine the detailed reasons for these differences, the oscillations uncovered in Fig. 2 ▸ may point to the plane-wave nature of the basis set. Whether this is actually the case remains to be investigated. All in all, the very compact nature of the density in ionic solids like NaCl leads to rather reproducible densities. This situation changes as covalency sets in.

## 3. Urea

Due to a number of reasons, the urea crystal has become one of the favorite systems for quantum crystallographers. It brings together the availability of high-resolution [sin(θ)/λ = 1.44 Å^−1^] X-ray data, as obtained by Birkedal *et al.* (2004 ▸), and a mixture of strong covalent CO and CN bonds in a hydrogen-bonded lattice characterized by a large crystal-induced dipole moment enhancement. It is thus a must in any QC-related discussion.

**Figure 3:**
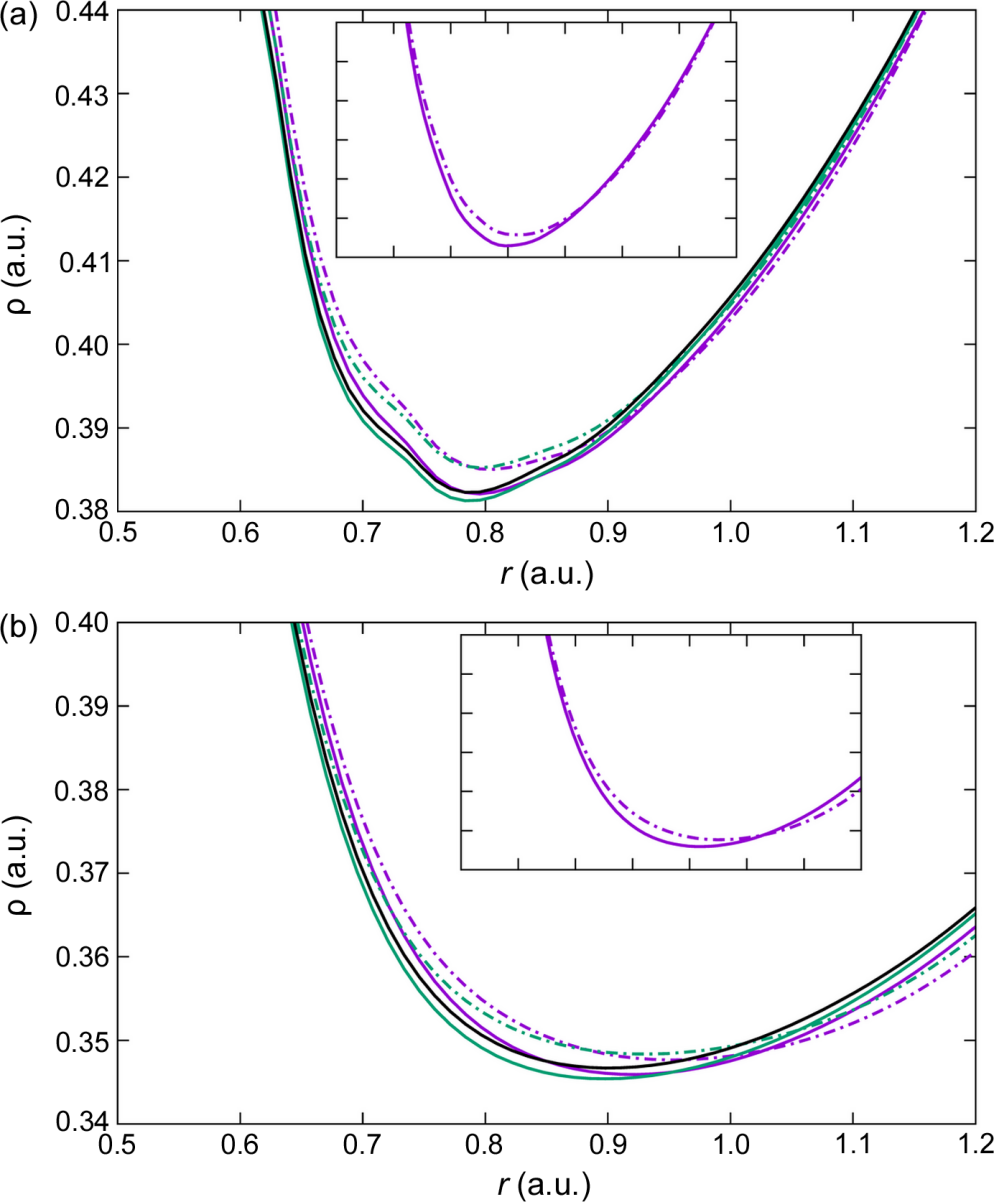
Electron densities as calculated with *VASP* (full lines) and *QE* (dashed lines) along the C—O (top) and C—N (bottom) internuclear axes in the urea crystal. Different DFAs are color coded: LDA in purple, PBE in green and PW91 in black. PW91 *QE* calculations did not converge with the specified default parameters. Distances are measured from the left nucleus of each pair. The wiggles along the CO line are an artifact from an insufficiently fine default FFT mesh (see the main text). As shown in the insets, where only LDA data are shown (see the main text for details), this pathology is solved using a finer mesh.

Fig. 3 ▸ displays the densities along the CO and CN bond lines. Before commenting on the density differences, the appearance of a set of spurious wiggles along the C—O internuclear axis is observed from both the *VASP* and the *QE* calculations. This is a clear indication of an insufficently saturated FFT grid that can easily go unnoticed to the researcher if only critical point data are collected. A PREC = Accurate *VASP* input leads to a (96, 96, 80) grid. Enlarging it to (160, 160, 128) solves the problem, as shown in the insets of Fig. 3, which display the larger grid LDA calculations. Aside from smoothing out the wiggles, the overall behavior of all finer-grid calculations remains untouched. Interestingly, the C—N bond axis is much less affected by this problem. This behavior is known and has been reported (Henkelman *et al.*, 2006 ▸; Yim & Klüner, 2008 ▸; Tang *et al.*, 2009 ▸). Here we caution the community against blindly using supposedly precise settings in electronic structure codes for QC applications. In times of high-throughput experiments and computations, it is not uncommon to bypass convergence tests on properties other than energy. Codes then become black boxes, providing a stream of data that is fed directly into spreadsheets or tables for further analysis. In Sections S6–S9 of the supporting information, we show that perfectly energy-converged calculations may not be density-converged, and that it is by far the size of the FFT grids that needs to be monitored in this regard. Although automatic procedures can be envisaged to detect the presence of density wiggles, their avoidance still depends on visual inspection. Also note that, unexpectedly, the PBE/*QE* calculation did not converge either with standard convergence settings or when changing the diagonalization algorithm. No problems were found, however, with the PW91 DFA. We have not investigated this issue in depth, but it definitely shows how the specific implementation of the algorithms available in the two codes can impact the results. Fig. 3 ▸ therefore lacks PBE/*QE* data.

The considerable electronegativity mismatch between carbon and either oxygen or nitrogen leads to an electron flow that complicates the analysis with respect to that in NaCl. Along the C—O line, for instance, and in the region surrounding the BCP, LDA provides larger densities than either PBE or PW91 in the carbon region, as in NaCl, but this behavior is inverted past the BCP. This inversion is rather common in covalent yet very heteropolar bonds. As also expected from theoretical grounds, now PBE and PW91 differ substantially more from each other than in the closed-shell NaCl crystal and, with regards to code dependency, *VASP* yields consistently smaller values than *QE* in the carbon region with a tendency towards inversion of this behavior in the oxygen basin. This was also found in the cationic and anionic regions along the Na—Cl, Cl—Cl and Na—Na bonds and could point to a systematic code dependency. Nevertheless, now the inter-code differences become larger than those associated with changes in DFAs. For instance, at the CO BCP the difference between the *QE* and *VASP* LDA densities is 2.8 × 10^−3^ a.u., whereas that between the *QE* PBE and LDA calculations is an order of magnitude smaller, 2.1 × 10^−4^ a.u. The same overall behavior is found along the CN direction, including the inversion of the LDA–GGA order as we shift from the cationic-like carbon region to the anionic-like nitrogen basin.

Table 1 ▸ shows the densities and Laplacians at BCPs for urea. Some critical points were not correctly identified along the nitrogen and distal hydrogen (H_d_) in the *VASP*/PBE calculation. A quick examination reveals that the weak O—H hydrogen bonds are much less sensitive to DFAs and codes than the strong covalent C—O and C—N interactions or even the N—H polar links. Although differences in densities between PBE and PW91 are smaller than between LDA and any GGA, the Laplacians display larger variations. Here the mismatch between codes becomes surprising. For instance, at the CO BCP the *QE*/PBE Laplacian (0.40 a.u.) is more than 2.5× smaller than in *VASP* (1.04 a.u.). However, no sign inversion in the Laplacians has been detected. Shared- or closed-shell interactions seem to maintain their character quite robustly.

A relevant point can be made in regards to the small changes in the position of the BCPs. Although relatively small, it affects the position of the quantum theory of atoms in molecules (QTAIM) interatomic surfaces, thus impacting the integrated electron populations and the atomic charges. We will not dwell on details here, since this has recently been considered by some of the present authors (Genoni & Martín Pendás, 2024 ▸). In the present context, for instance, a smaller carbon atom along the C—N line in *QE*/LDA with respect to *VASP* (0.95 versus 0.92 a.u., respectively) means a non-negligible code-related dependence of the carbon electron population.

Since default computational conditions may lead to not fully converged densities which can also affect the position of critical points, it is interesting to check whether the different densities or Laplacians in Table 1 ▸ are due to the BCP position. We have thus computed these scalars with different computational conditions at fixed positions in space in Sections S7–S9 of the supporting information. As shown, code differences remain. Although both ρ and 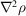 evolve smoothly at the chosen fixed point on the CN line, the Laplacian oscillates between positive and negative values in the CO case. This demonstrates how sensitive these values can be in very polar bonds where the BCP tends to be located close to a zero of 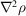.

**Figure 4:**
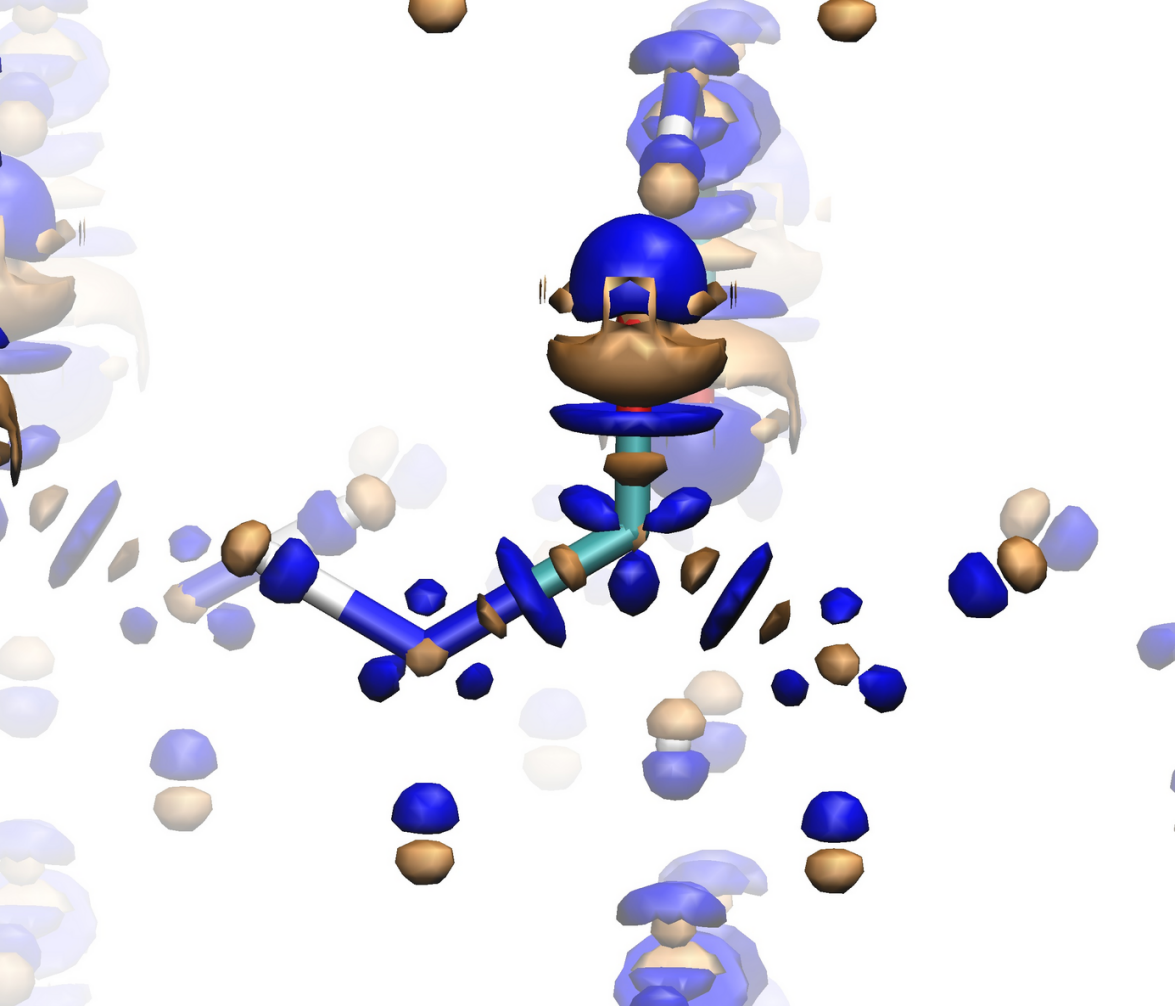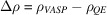 difference density maps for urea as obtained with the LDA DFA with 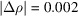 a.u. Positive and negative isosurfaces are depicted in blue and orange, respectively. A central urea molecule is highlighted.

Fig. 4 ▸ shows the 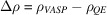 = ±0.002 a.u. isosurfaces obtained with LDA. A similar distribution is obtained with PBE, shown in the supporting information (Fig. S2). First, let us stress that, as we move outside the nuclei, we observe the same oscillatory behavior that was already found in the quasi-spherical NaCl density distribution. Again, whether this is due to the plane-wave nature of the basis set is still to be ascertained. It is nevertheless interesting that a clearly repetitive pattern is observed. Both in the LDA and in the GGA descriptions (see the supporting information), the inner core regions receive more density in the *QE* calculation and *VASP* tends to accumulate density in the outer cores, outside the bond axes (see the blue triangular-shaped features around the nitrogen and carbon atoms). Along the bond axes a peculiar oscillating distribution is also ascertained. As we move from carbon to nitrogen along a C—N bond, for instance, regions where 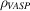 is smaller, then larger, then smaller again than 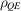 follow one another in a rather symmetric way. Noticeably, in the central bond region the *VASP* code tends clearly to accumulate density. We also notice that in the weird symmetry lies an indication of the non-chemical nature of these differences, since the BCPs around the carbon atom are far from the center of any of the C—(N, O) internuclear lines, closer to the three triangular orange blobs observed in the figure. Also to be noted is the dipolar nature of the differences around the hydrogen atoms, *VASP* accumulating density with respect to *QE* along the N—H lines and the more important differences found in the oxygen basin, with *VASP* providing a larger density in the lone-pair regions. As far as non-covalent hydrogen-bond regions are concerned, it is *QE* which accumulates density around them both in the hydrogen and in the oxygen domains (see the four small orange surfaces surrounding the oxygen lone pairs).

## 4. SbH_4_: how the density retrieved can lead to different property estimations

It has recently been shown that electron delocalization, as provided by the electron localization function (ELF) (Becke & Edgecombe, 1990 ▸; Silvi & Savin, 1994 ▸), can be related to the critical temperature in hydrogen-based superconductors (Belli *et al.*, 2021 ▸). Indeed, the value of the ELF for which a 3D-connected surface is obtained (*i.e.* networking value or ϕ) allows us to cast the delocalization over a solid, and it has been shown to correlate with the critical temperature (*i.e.* the metallicity) of hydrogen-based superconductors. However, hydrogen atoms being specially diffuse, these measures can be especially prone to software dependency. We will showcase this with the *P*6_3_/*mmc* phase of the SbH_4_ system, which has been predicted to be a superconductor. It features both covalent and ionic bonds, staying metallic in the 127–300 GPa range. This mixture of bonding properties makes it most interesting for our study, especially taking into account that superconductivity is predicted with a *T*_c_ of 106 K at 150 GPa. Since Belli’s approach was introduced for *QE* calculations, here we will test whether new fittings are needed for *VASP* calculations.

**Figure 5:**
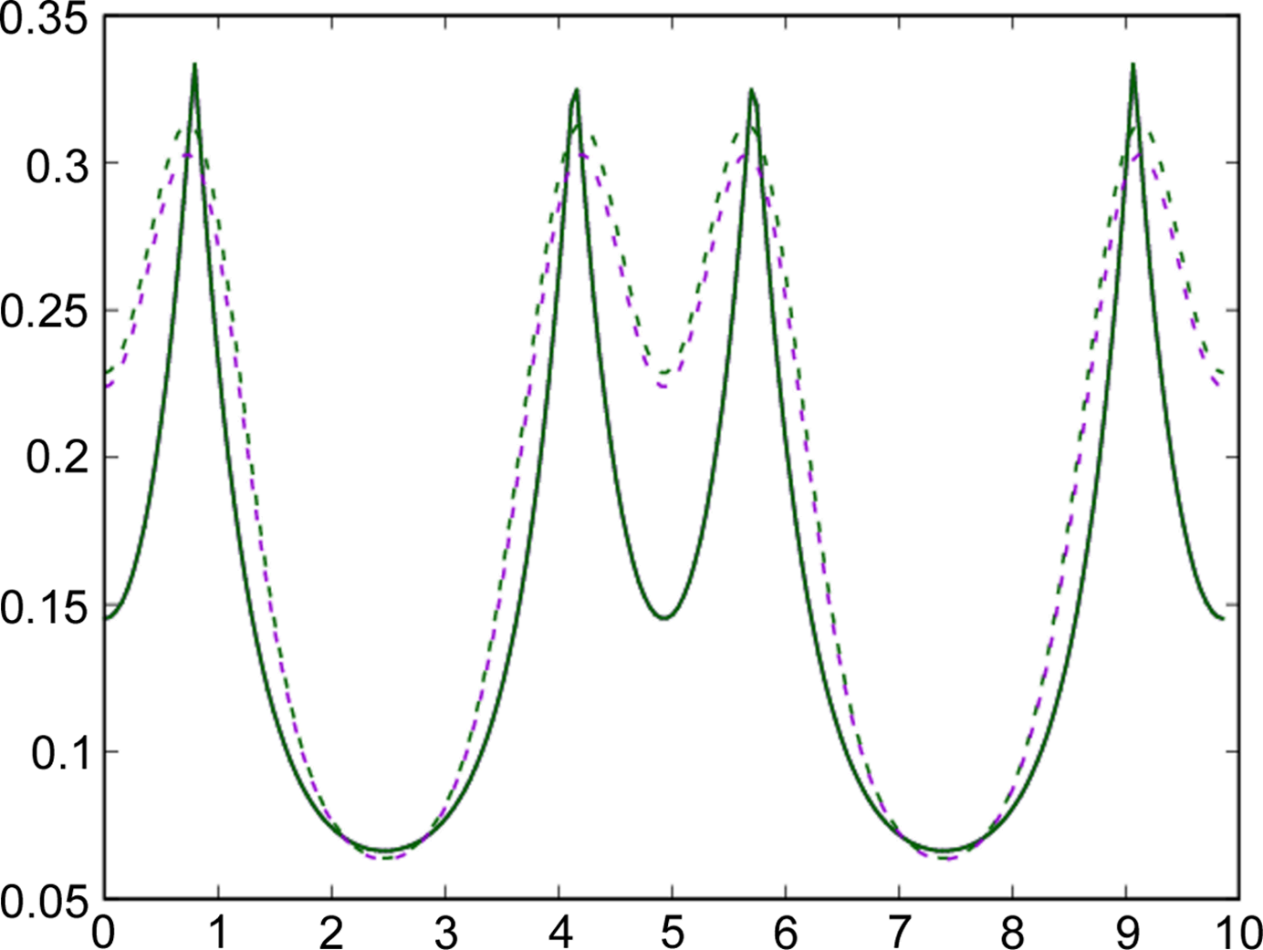
Electron densities as calculated with *VASP* (full lines) and *QE* (dashed lines) along the *z* axis in the SbH_4_ crystal. Different DFAs are color coded: LDA in purple and PBE in green. Note that LDA and PBE lines overlap within the *VASP* calculation.

Hydrogen-based superconductors lead to delocalization networks mainly centered on hydrogens, so we have focused on analyzing hydrogen interactions. Fig. 5 ▸ shows the electron density computed along the *z* direction, which contains one H_2_ molecule in the middle of the axis as well as two H⋯H contacts with neighboring molecules at the border of the cell. The cell parameters have been taken from Fu *et al.* (2016 ▸).

Results are shown for the LDA and PBE functionals with the *QE*- and *VASP*-suggested PBE pseudopotentials. Results for PW91 are not shown since the aim here is to compare LDA with GGA, which have shown the greatest disagreements. No differences are observed for *VASP*.

We see that important differences appear between the *VASP* and *QE* implementations. The differences are clear for the H—H molecular bond, where the BCP shows a much greater density in *QE* (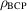 = 0.2286 a.u.) than with *VASP* (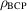 = 0.1452 a.u.). Moreover, the Laplacian at this point, though negative in both cases, is much bigger in absolute value in the *QE* calculation than in the *VASP* one. In other words, the bond is much more covalent in *QE*.

**Figure 6:**
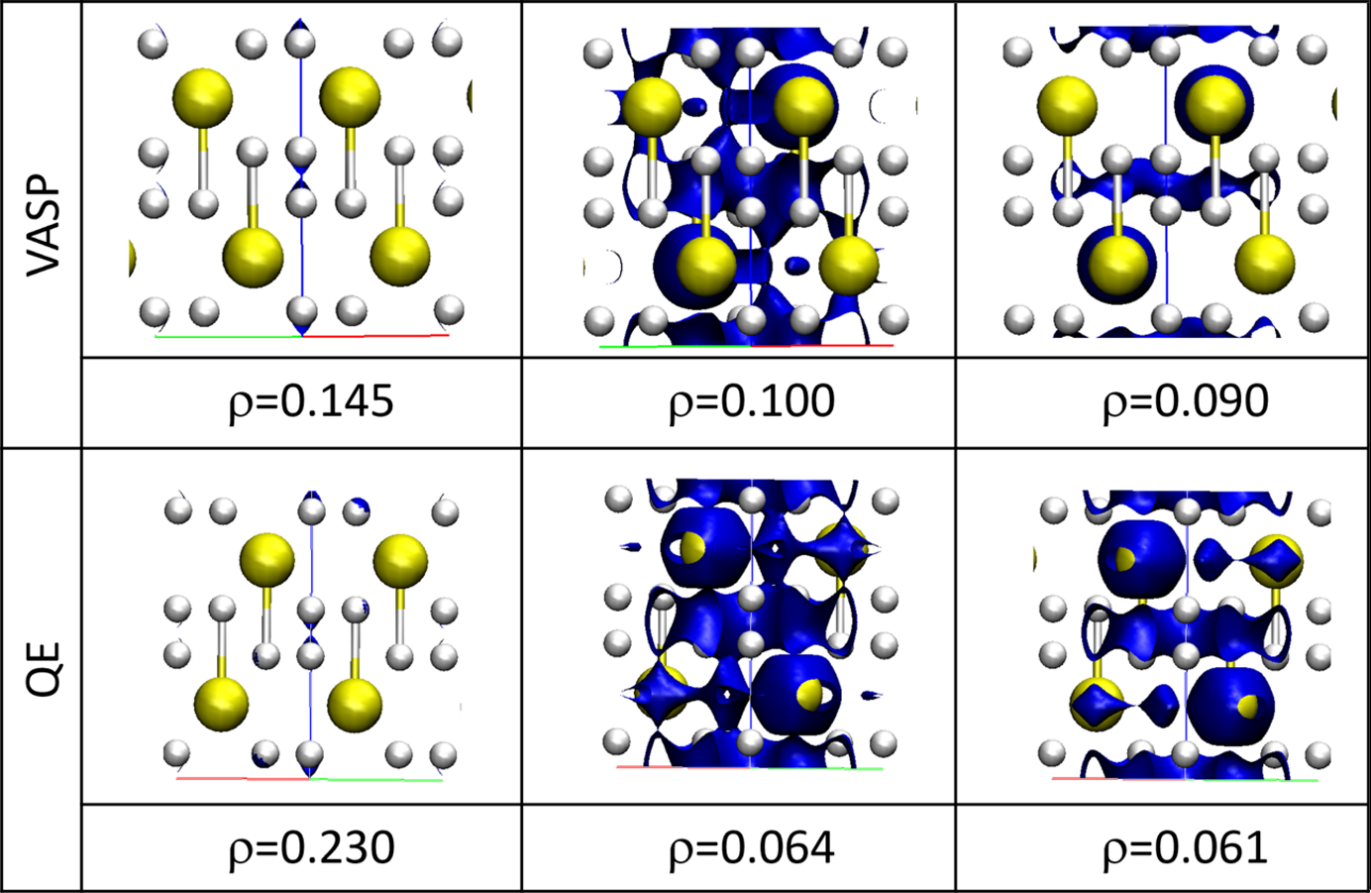
Electron density isosurfaces at the BCPs selected in Table 2 ▸. Data are given in a.u.

The overall shape of the electron density is also affected. Critical points within the intermolecular region appear at different places (see Table 2 ▸, and also Tables S3 and S4) leading to different bonding patterns among the different clathrate units. To illustrate this, the isosurfaces of ρ at the main BCP values are collected in Fig. 6 ▸. Differences appear not only in the shape of the heavy atom but also in the isosurfaces at low densities.

**Figure 7:**
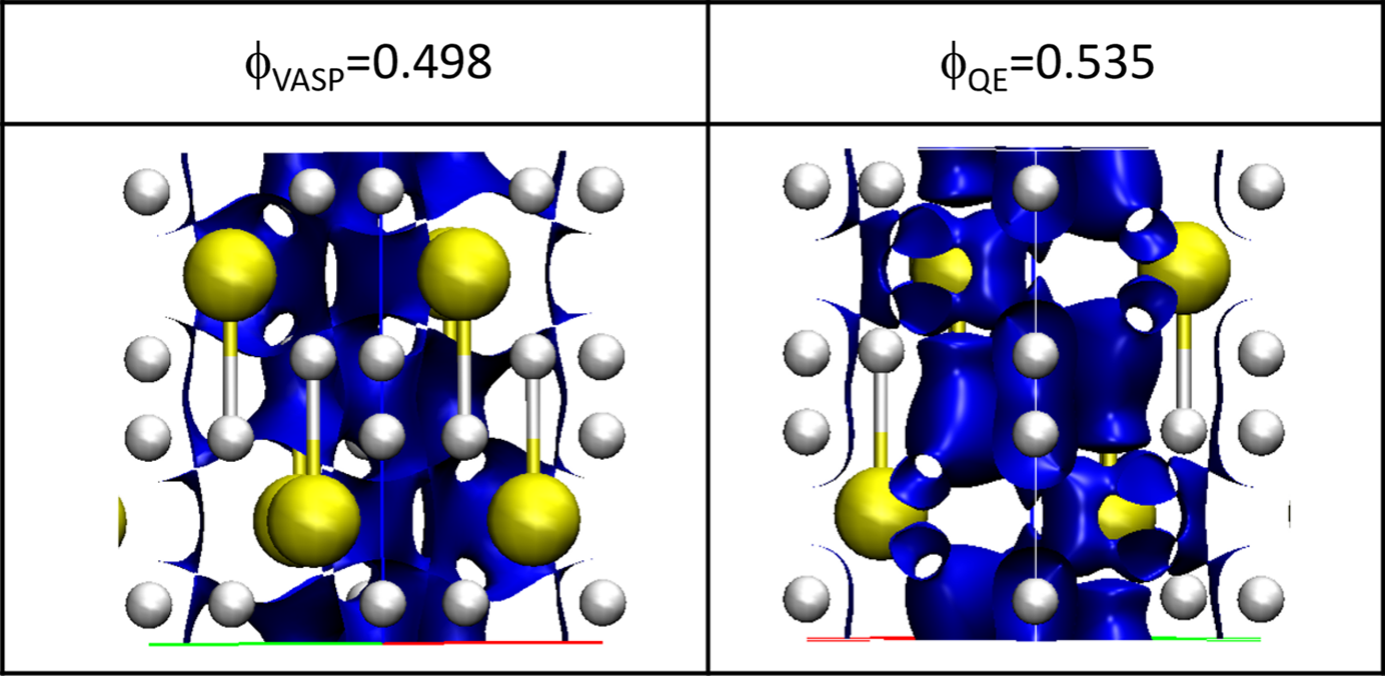
Networking value isosurfaces (Belli *et al.*, 2021 ▸) (as defined in the text) for *VASP* and *QE*.

Since low-density regions are expected to be related to the delocalization of electrons through the cell, which correlates with the critical temperature of the solid, we have also plotted the ELF and looked for the so-called networking value (at which we obtain a full delocalization through the cell) (Belli *et al.*, 2021 ▸). These are shown in Fig. 7 ▸. As expected, different values are obtained with the two codes [ϕ = 0.498 with *VASP* and ϕ = 0.535 with *QE*, where ϕ represents the electron localization value at which the surface is 3D connected (Belli *et al.*, 2021 ▸)].

The point that we want to highlight here is that these differences are due to neither the functional nor the pseudopotential. An important consequence of the observed variations is that correlations for the solid state with the aim of predicting macroscopic properties are probably now software-dependent.

## 5. The MgM crystal

As mentioned, the MgM system shows an RAHB (Gilli *et al.*, 1989 ▸; Bertolasi *et al.*, 1991 ▸; Gilli *et al.*, 1994 ▸) within the maleate anion (Fig. S1). From a combination of QTAIM and energy-decomposition analysis, it was concluded that this type of strong hydrogen bond is stabilized by electron delocalization and possesses a covalent (or partially covalent) nature (Grabowski, 2003 ▸; Gora *et al.*, 2005 ▸). Nevertheless, the examination of the components of the interaction energy defined within the interacting quantum atoms approach (Blanco *et al.*, 2005 ▸) revealed that the stabilization comes mainly from the classic electrostatic component (Guevara-Vela *et al.*, 2016 ▸). Thus, this type of system is challenging because it appears that the RAHB lies on the border between a covalent and an ionic bond.

**Figure 8:**
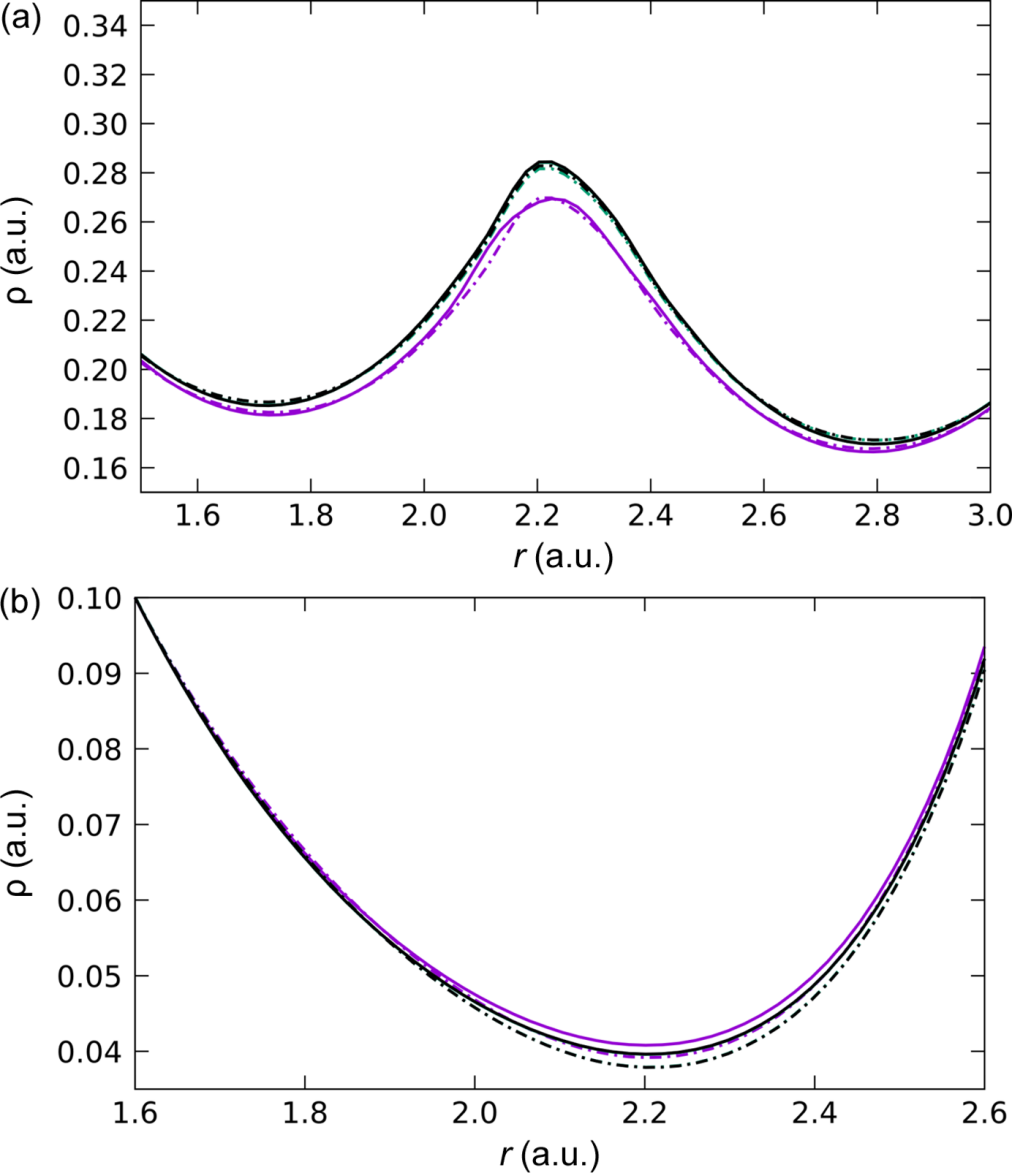
Electron densities as calculated with *VASP* (full lines) and *QE* (dashed lines) along (*a*) the strong resonance-assisted intramolecular [O1⋯H⋯O2]^−^ hydrogen bond and (*b*) the O3
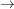
Mg coordination bond. Similar behavior was found for the other two dative bonds. Different DFAs are color coded: LDA in purple, PBE in green and PW91 in black.

**Figure 9:**
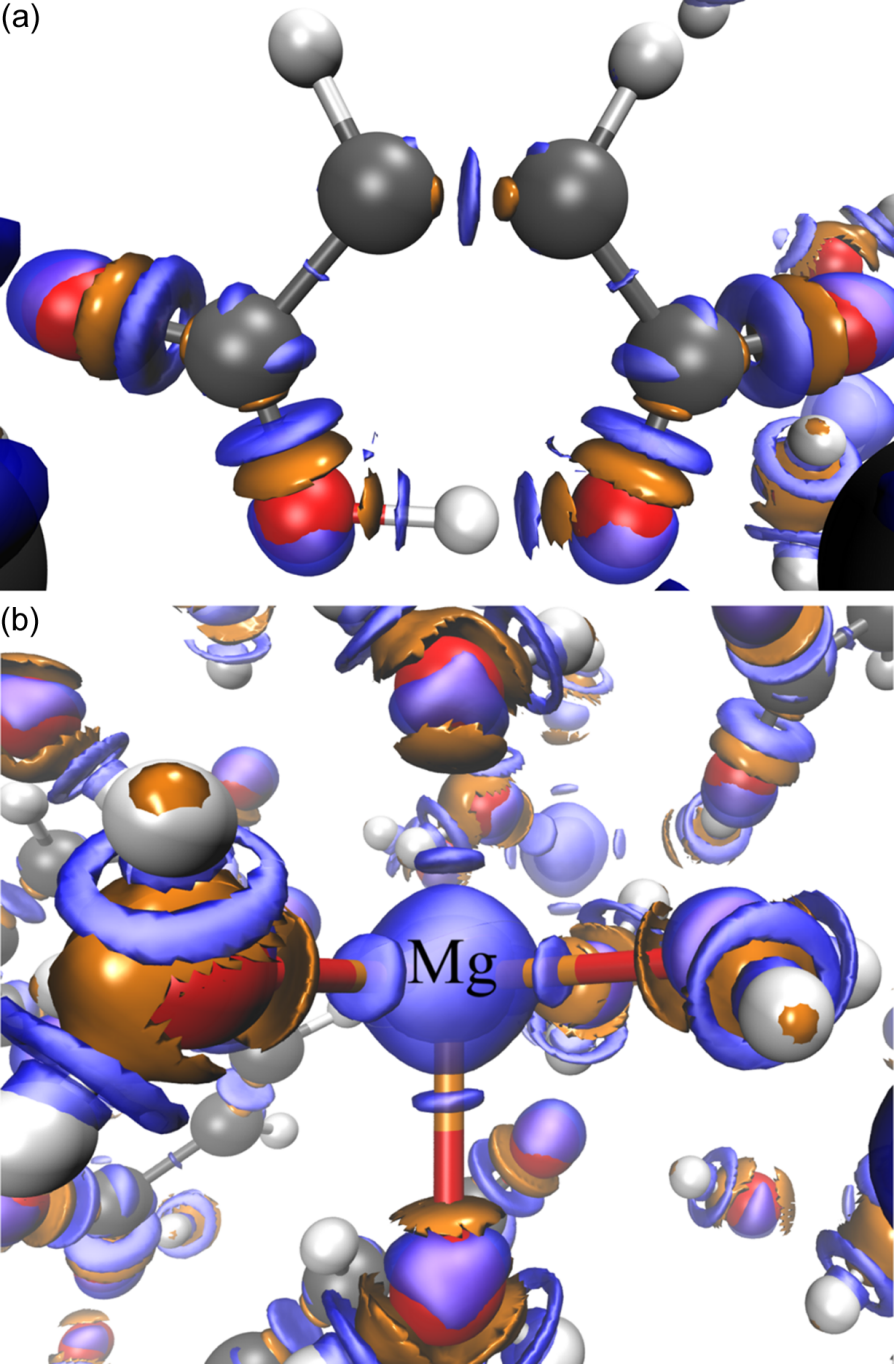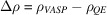 difference density maps for MgM as obtained with the PBE DFA (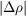 = 0.0025 a.u.) in (*a*) the maleate anion and (*b*) the [Mg(H_2_O)_6_]^2+^ complex. Positive and negative isosurfaces are depicted in blue and orange, respectively.

Fig. 8 ▸(*a*) displays the electron density along the line connecting the two oxygen atoms (O1 and O2) that participate in the RAHB. Although the hydrogen-atom nucleus does not lie exactly along this line, it is very close to it, and the most important features of the behavior of the electron density at the nucleus can still be appreciated. The first thing to note is that both GGA functionals, with both codes, provide virtually identical results. On the other hand, the LDA DFA provides lower values of electron density in the vicinities of the nucleus and the BCP with both codes. The small difference between 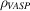 and 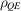 observed in the left part of Fig. 8 ▸(*a*) can be attributed to the deficiency in the fine-FFT grid discussed before. Also, the electron density distribution on the two sides of the hydrogen atom is not symmetric, since the atom is not placed exactly at the middle of the two oxygen atoms. The corresponding O1⋯H⋯O2 bond lengths are 1.181 and 1.226 Å, respectively. Another important observation is that, just like when using Gaussian basis sets, plane waves do not reproduce the cusp of the electron density at the nuclear position. This can be corroborated by analyzing the electron density along the H—O1 bond (Fig. S3), where it is more evident that Kato’s theorem is not fulfilled. It has been recently shown that correctly taking this phenomenon into account could improve the crystallographic refinements (Kleemiss *et al.*, 2024 ▸). Given how problematic the refinement of hydrogen atoms is in some cases, this fact could be taken into account in future work to improve this issue when using solid-state codes. In addition, the electron density lines of GGA and LDA are found to become closer the further away they are from the nucleus. Indeed, they will eventually cross over, and those of different codes will generate oscillations similar to those described above for the covalent bonds in urea. For instance, the oscillation can be observed in the PBE 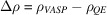 difference density map [Fig. 9 ▸(*a*)]. This pattern is more pronounced in the C—O covalent bonds, as well as in the regions around the oxygen atom where the lone pairs are expected to be. In contrast, 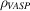 and 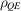 show a very similar behavior in the C—C bonds.

In the case of the O
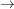
Mg bonds, there are noticeable differences in the electron density near the BCP [Fig. 8 ▸(*b*)]. Now it is PBE(*VASP*), PW91(*VASP*) and LDA(*QE*) that show a similar behavior. LDA(*VASP*) shows higher ρ values near the BCP, and PBE(*QE*) and PW91(*QE*) display lower values. Therefore, for this type of interaction, the influence of the code seems to be as relevant as that of the DFA. Far from the BCP the lines will also intersect. The PBE 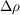 = 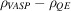 difference density map in the [Mg(H_2_O)_6_]^2+^ complex is shown in Fig. 9 ▸(*b*). The blue zones, where the *VASP* density predominates, are almost spherical around the Mg^2+^ cation together with some discs.

On the other hand, the *QE* density predominates (orange surfaces) in a region bordering the oxygen atoms of the water molecule, near the O—H bonds, also with an orange disc appearing in the region corresponding to the coordinate bond, closer to oxygen. As one gets even closer to the oxygen nucleus in the direction of the dative bond, again an oscillation appears where the *VASP* density dominates. Furthermore, there also appear rings of *VASP* ρ accumulation around the O—H covalent bonds of the water molecules. At least for the isosurface selected in Fig. 9, no appreciable code dependence was observed for the hydrogen bonds formed between the water molecules and the maleate anion.

The analysis of the BCPs turned out to be more troublesome. To make a fair comparison between the results of both codes, the search for critical points was performed on electron density cubes in a 2 × 1 × 2 supercell, with a space grid equal to that of the fine-FFT. Except for PW91(*QE*), only one of the two expected BCPs of the RAHB could be located with both codes and all the DFAs (Table S5). A further exploration revealed that small changes in the electron density cube grids (for instance, changing the number of points in one of the axes from 336 to 335) cause the search to find both BCPs or none. This highlights another general problem of density analyses with plane-wave solid-state quantum mechanical codes: neither the topological analysis nor the charge integrations performed with grid-based techniques is as straightforward as in molecular calculations, where analytical derivatives using WFN or WFX files are available. This has been considered many times (*e.g.* Yu & Trinkle, 2011 ▸; Otero-de-la Roza, 2022 ▸). Since it is not our aim to provide accurate topologies or densities here, but to highlight how different they can be when using different codes, we will not pay more attention to this. We nevertheless note that grids can of course be fully avoided using local basis sets from which analytical derivatives are available. This approach has been implemented, for instance, in the *CRYSTAL* code (Erba *et al.*, 2023 ▸) through its *TOPOND* package, originally written by Carlo Gatti. Note that local basis sets pose their own convergence problems, which are difficult to relate to those of plane waves. The difference in 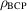 among the various DFAs and both codes is relatively small (around 2%). All the 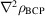 values are negative, consistent with a covalent bond. However, the difference among the 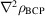 values obtained with various DFAs is, on average, 4%, while it can be as large as 23% between two codes with the same DFA. Comparable results were found for the analysis of the BCPs corresponding to the coordination bonds in the [Mg(H_2_O)_6_]^2+^ complex (Table S6). Of the three symmetry non-equivalent O
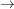
Mg interactions, only two were found with LDA(*QE*) and PBE(*QE*). No issues were encountered with the remaining combinations of DFAs and codes. The difference in 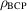 between the different codes was on average only slightly larger than in the RAHB case (4%), but increased to around 30% for 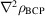. Thus, it is corroborated that using different electronic structure codes will have an impact on the topological analysis of the electron density. The Laplacian, especially in the case of non-covalent interactions such as coordination bonds, appears to be particularly sensitive to this.

## 6. Conclusions

Although typical electronic structure calculations have focused on the variational minimization of the energy, it has become increasingly clear that the behavior of the electron density should not be ignored in calculations. This point has been made by Medvedev *et al.* (2017 ▸) after showing convergence of the former but not of the latter in molecular DFT calculations. However, not much is known about similar problems in condensed phases, which can become more important in the coming years, especially considering that other accurate quantum mechanical methods to compare with will typically still be out of reach.

The coming of age of quantum crystallography promises a period of intense comparison between theoretically calculated and experimentally determined electron densities. Therefore, it is opportune to examine in detail all the aspects that may influence this comparison. One point that has gone unnoticed so far is the possible dependence of the calculated densities on the electronic structure code used to obtain them. As demonstrated in this work, this may not be negligible, so we recommend that this factor be taken into account.

Here we analyzed that dependence by selecting the projector augmented wave method implemented in the *QE* and *VASP* codes. First, we chose two systems that constitute milestones and references in crystallography: NaCl and urea crystals. Our results show that very ionic crystals, represented by NaCl, give densities that depend very little on the code. However, differences start to appear when analyzing covalent and non-covalent interactions. Such is the case for urea, where several non-negligible differences between *QE* and *VASP* have been observed even when using computational conditions as coincident as possible in both codes.

Firstly, insufficient FFT grids lead to density wiggles that appear preferentially in *QE* rather than in *VASP*. These are eliminated if a larger FFT grid is selected. Note, however, that the computational time for each of the two codes varies considerably. For instance, the CPU times for NaCl with the coarse grid using *QE* and *VASP* were 45 and 16298 s, respectively. Increasing the fineness of the FFT grid in *QE* to remove the wiggles increases the computing time to 128 s. Therefore, quantum crystallography users and developers are advised to check electron density profiles before production calculations to judiciously choose grid sizes.

Small dependencies on the chosen density functional are also observed that typically follow expected trends from the delocalization error. In this regard, users should be aware of the general trends: LDAs will typically lead to more delocalized (flatter) distributions.

Finally, and probably the most important point to be highlighted from these results, is the up-to-now unnoticed software dependence. Significant differences have been observed between *QE* and *VASP*, which increase with the complexity of the system. *QE* tends to accumulate charge in the inner core regions, whereas *VASP* tends to do so in the outer cores, outside the bond axes. To demonstrate that this is not a result peculiar to the urea crystal we have examined two other systems, a high-pressure superconducting phase of antimony hydride and the magnesium bis(hydrogen maleate) hexahydrate crystal (MgM), which shows covalent, ionic and dative interactions in a single system.

In the first case, it has previously been shown that electron delocalization can be used to estimate the superconducting critical temperature. The present results show that this estimate should even be calculated with different correlation expressions depending on the code used. Hence, we expect that other properties, such as binding energies, also require the specification of the code used. Finally, the analysis of MgM revealed some additional features. Despite using a large set of wave planes, the cusp condition near the hydrogen-atom nucleus is far from being fulfilled. The code dependence has a greater impact on coordination than on covalent bonds, especially when analyzing the Laplacian of the electron density, for which the difference in the values at the BCP (when using the same DFA) can be as large as 30%.

Overall, we would like to emphasize the importance of analyzing the stability of the electron density computed from periodic codes, paying attention to the FFT grid and the code used. We believe that further studies of the code dependence of electron densities unveiled here will be needed for future implementations in the framework of quantum crystallography.

Since automated procedures to mitigate these problems are conceivable in principle, but not yet available, we recommend a simple strategy in the meantime. Whenever densities and their topologies are obtained with plane-wave codes and are considered important for a given topic, a visual inspection of the evolution of the density along very polar and/or one-electron-dominated regions under varying computational conditions should be a must.

## Supplementary Material

Supporting figures and tables. DOI: 10.1107/S2052252525001721/woz5007sup1.pdf

## Figures and Tables

**Table 1:** Densities, Laplacians and bonded radii at relevant critical points in the urea crystal Bonded radii correspond to the left-most atom in the pair. Labeling follows that in Fig. S1 and the data are given in a.u.

	*QE*/LDA	*QE*/PBE	*VASP*/LDA	*VASP*/PBE	*VASP*/PW91
CO	0.38503	0.38523	0.38210	0.38125	0.38225
CN	0.34766	0.34835	0.34591	0.34540	0.34669
ρNH_d_	0.33857	0.34762	0.34363	–	0.35251
ρNH_g_	0.33562	0.34457	0.34071	0.34929	0.34953
ρOH_g_	0.02318	0.02264	0.02325	0.02270	0.02278
ρOH_d_	0.02018	0.01954	0.02022	0.01957	0.01964
∇^2^ρ(CO)	0.05695	0.40403	0.70625	1.04327	0.97923
∇^2^ρ(CN)	−1.11265	−1.09210	−0.96025	−1.10297	−1.06425
∇^2^ρ(NH_d_)	−2.04545	−1.70949	−1.81598	–	−1.83991
∇^2^ρ(NH_g_)	−1.78188	−1.59391	−1.49108	−2.59501	−2.64536
∇^2^ρ(OH_g_)	0.07318	0.07656	0.07379	0.07234	0.07235
∇^2^ρ(OH_d_)	0.07084	0.07220	0.06895	0.07056	0.07054
*r*(CO)	0.80340	0.79560	0.79410	0.78740	0.78820
*r*(CN)	0.95260	0.92940	0.92020	0.89780	0.90130
*r*(NH_d_)	1.47920	1.46120	1.45140	–	1.43320
*r*(NH_g_)	1.48650	1.47040	1.46170	1.44730	1.44580
*r*(OH_g_)	2.43100	2.42680	2.43070	2.42610	2.42610
*r*(OH_d_)	2.46220	2.45710	2.46220	2.45770	2.45770

**Table 2:** Selected critical point positions, electron density and Laplacian for SbH_4_ as obtained with *VASP* and *QE* H—Sb—H (*x*) represents the H—Sb—H interaction along the *x* axis and so on. We have highlighted in bold the electron density value at which a ρ isosurface running all through the cell is obtained. All data are given in a.u.

		H—H intramolecular	H—Sb⋯H (*x*)	H—Sb⋯H (*z*)
*VASP*	(*x*, *y*, *z*)	(0, 0, 0)	(2/3, 1/3, 0.526)	(0.113, 0.887, 0.362)
	ρ, ∇^2^ρ	0.14516, −0.07193	**0.09832**, 0.11950	0.08861, 0.11214

*QE*	(*x*, *y*, *z*)	(0, 0, 0)	(0.327, 0.164, 0.122)	(2/3, 1/3, 1/4)
	ρ, ∇^2^ρ	0.22857, −0.86902	**0.06402**, 0.11165	0.06098, 0.12522
