# Supplementary material for: Code dependence of calculated crystalline electron densities. Possible lessons for quantum crystallography
Source: IUCrJ. 2025 Mar 28;12(Pt 3):295–306. doi: 10.1107/S2052252525001721 (PMC12044850; doi:10.1107/S2052252525001721)
Supplement: Supplementary file 1 [file m-12-00295-sup1.pdf]

IUCrJ

Volume 12 (2025)

Supporting information for article:

Code dependence of calculated crystalline electron densities. Possible lessons for Quantum Crystallography.

Bruno Landeros-Rivera, Julia Contreras-García and Ángel Martín Pendás

Supporting Information.
Code dependence of calculated crystalline electron densities. Possible lessons for Quantum Crystallography.

Bruno Landeros-Rivera,[c] Julia Contreras-García[b] and Ángel Martín Pendás [a]*

[a]*Dept. Química Física y Analítica, Universidad de Oviedo, 33006 Oviedo, Spain,*
[b]*Laboratoire de Chimie Théorique, Sorbonne Université, F. 75005 Paris, France,*
and [c]*Dept. Química Inorgánica y Nuclear, Universidad Nacional Autónoma De México, 04510 Ciudad de México, México. E-mail: ampendas@uniovi.es*

All data in the supporting information is expressed in atomic units.

S1. Crystallographic data and computational details

Table S1. *Crystallographic data, K-points, FFT and fine-FFT mesh size.*

System	NaCl	Urea	SbH_4	MgM
Space group	Fm-3m	P-42(1)m	P6(3)/mmc	P2(1)/c
a/Å	5.58812644	5.57800007	2.984877	10.19200040
b/Å	5.58812644	5.57800007	2.984877	11.75599957
c/Å	5.58812644	4.68599987	5.560137	6.43167903
$\alpha/^\circ$	90	90	90	90
$\beta/^\circ$	90	90	90	103.66
$\gamma/^\circ$	90	90	120	90
Monkhorst grid	8 x 8 x 8	8 x 8 x 10	16 x 16 x 9	1 x 1 x 2
K points	35	90	1920	2[†]
FFT mesh	48 x 48 x 48	48 x 48 x 40	24 x 24 x 48	84 x 96 x 54
Fine FFt mesh	96 x 96 x 96	96 x 96 x 80	40 x 40 x 72	168 x 192 x 108

[†] Although the K-points mesh was generated using a 1x1x2 spacing, with the automatic Monkhorst-Pack uniform grid generator of each code, VASP generated only 1 K-point, while QE generated 2. Despite the former convergence tests showed that the difference between the two codes cannot be attributed to this, it draws attention that the generation of the same mesh of K points is not simple.

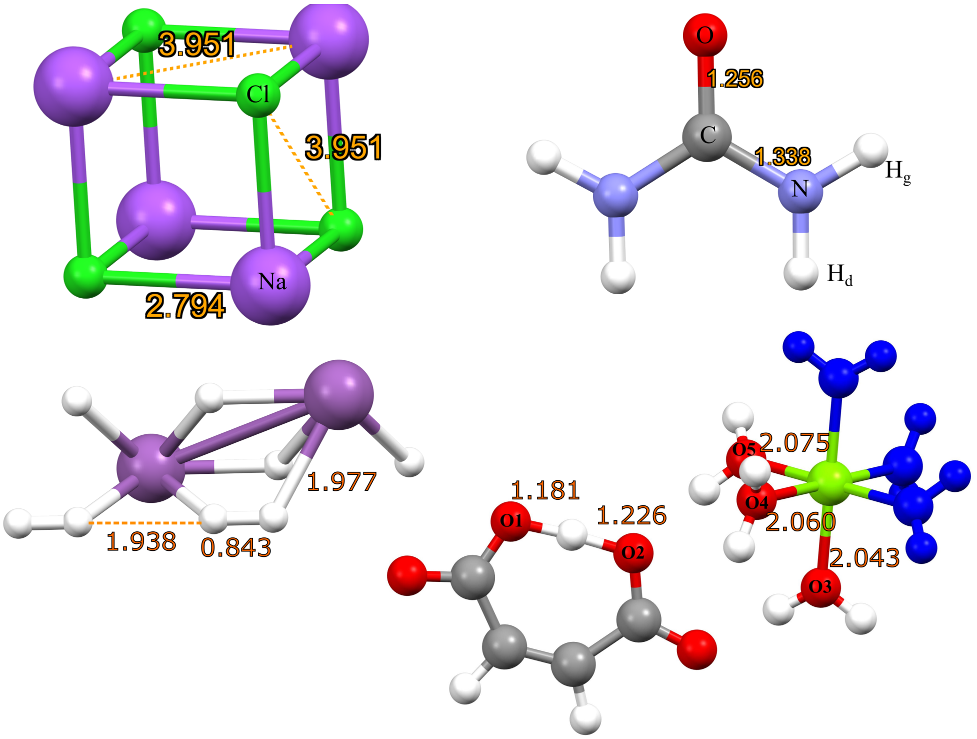

Fig. S1. Most relevant atom labeling and bonding distances (in Å) of the analyzed systems. The blue water molecules in MGA are symmetry equivalent.

S2. NaCl

Table S2. *Densities, Laplacians, and bonded radii at relevant critical points in the NaCl crystal. Bonded radii correspond to the Na atom in the NaCl pair*

	ρ-NaCl	ρ-ClCl	$\nabla^2\rho$-NaCl	$\nabla^2\rho$-ClCl	r(Na)
QE-LDA	0.01301	0.00528	0.08179	0.00141	1.95070
QE-PBE	0.01247	0.00499	0.08560	0.00122	1.94720
QE-PW91	0.01244	0.00499	0.08761	0.00245	1.94460
VASP-LDA	0.01307	0.00529	0.05779	0.01257	1.94620
VASP-PBE	0.01266	0.00500	0.05923	0.01260	1.94540
VASP-PW91	0.01263	0.00499	0.05777	0.01248	1.94800

S3. Urea

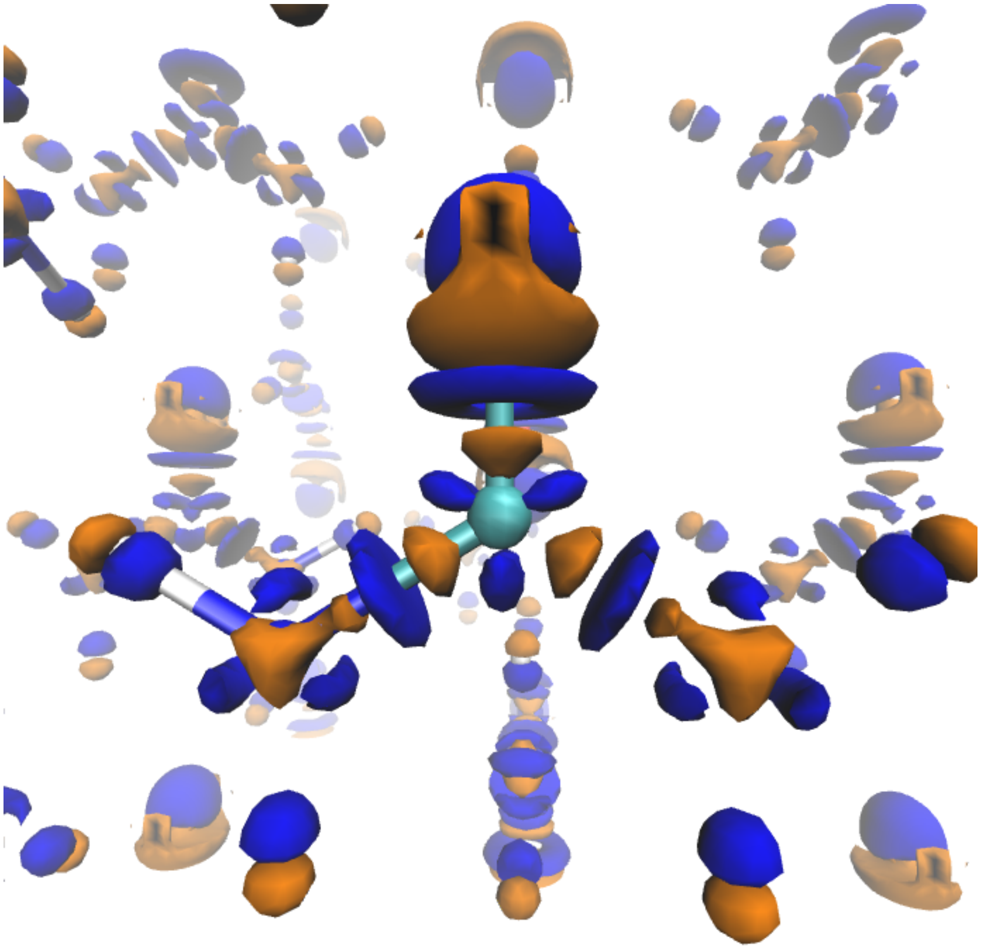

Fig. S2. $\Delta\rho = \rho_{\text{VASP}} - \rho_{\text{QE}}$ difference density maps for urea as obtained with the PBE DFA with $|\Delta\rho| = 0.002$ au. Positive and negative isosurfaces are depicted in blue and orange, respectively. A central urea molecule is highlighted.

S4. SbH_4

Table S3. *Crystallographic positions, multiplicities, densities, and Laplacian of the VASP PBE description of* SbH_4

BCP	x	y	z	mult	ρ	$\nabla^2\rho$
1	0.66849011	0.83424263	0.75000053	6	6.87572E-02	9.55746E-02
2	0.44446583	0.55553775	0.86078143	12	8.79415E-02	1.08714E-01
3	0.11288338	0.88711600	0.36241608	12	8.86157E-02	1.12145E-01
4	0.33333167	0.66666944	0.47362139	4	9.83236E-02	1.19503E-01
5	0.99999842	0.99999842	1.00000000	2	1.45164E-01	-7.19357E-02

Table S4. *Crystallographic positions, multiplicities, densities, and Laplacian of the QE PBE description of SbH_4*

BCP	x	y	z	mult	ρ	$\nabla^2\rho$
1	0.00000000	0.00000000	0.00000000	2	2.28570000E-01	-8.69021307E-01
2	0.12455915	0.85969282	0.25000000	12	7.00536423E-02	-1.29942720E-01
3	0.02604981	0.35237692	0.33481837	24	6.89716135E-02	-5.93522190E-02
4	0.67480532	0.02491162	0.66598255	24	6.89706529E-02	-1.08115538E-01
5	0.55126478	0.10269793	0.60342385	12	6.80096465E-02	-7.53968070E-02
6	0.53322264	0.06625604	0.25000633	6	6.76367025E-02	-5.20722204E-02
7	0.43076546	0.21558883	0.60748720	12	6.64048708E-02	-8.27423912E-02
8	0.32677425	0.16387630	0.12239567	12	6.40178497E-02	1.11652422E-01
9	0.00000000	0.00000000	0.25000000	2	6.37970000E-02	9.52505976E-02
10	0.66682456	0.33317544	0.25000007	2	6.09792511E-02	1.25225625E-01

S5. MgM

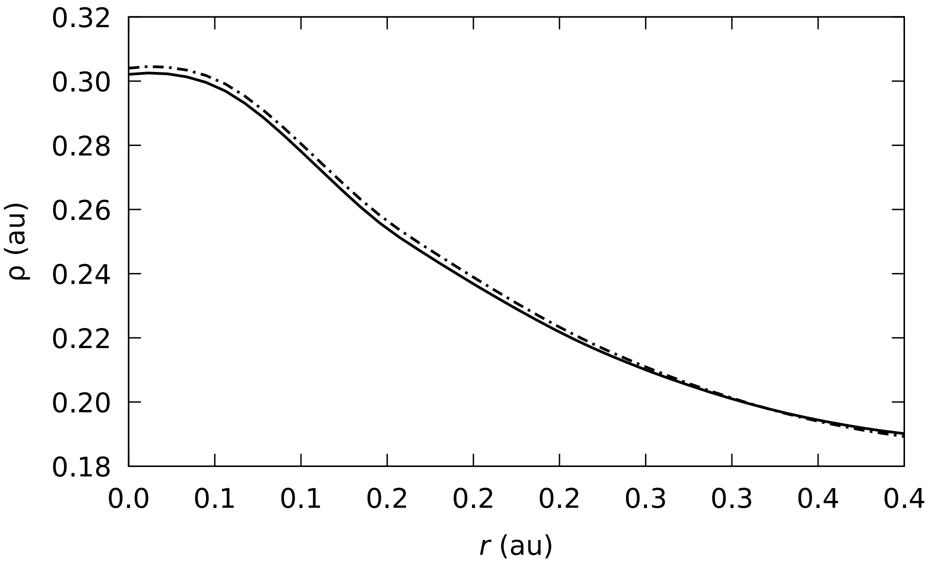

Fig. S3. Electron density along the H-O1 bond in MGM calculated with the PBE DFA, using VASP (continuous) and QE (dashed). Similar results were obtained for the other DFAs.

Table S5. *Densities and Laplacians (a.u.) at the two relevant critical points in the RABH of the maleate anion of MgM. NF stands for not found.*

	ρ-O1-H	$\nabla^2\rho$-O1-H	ρ-O2-H	$\nabla^2\rho$-O2-H
QE-LDA	0.16862	-0.43285	NF	NF
QE-PBE	0.17208	-0.41823	NF	NF
QE-PW91	0.17215	-0.41816	0.18785	-0.59705
VASP-LDA	0.16725	-0.35793	NF	NF
VASP-PBE	0.17054	-0.34012	NF	NF
VASP-PW91	0.17048	-0.34163	NF	NF

Table S6. *Densities and Laplacians (a.u.) at three symmetry non-equivalent O→Mg coordination bonds of MgM. NF stands for not found.*

	ρ-O3→Mg	$\nabla^2\rho$-O3→Mg	ρ-O4→Mg	$\nabla^2\rho$-O4→Mg	ρ-O5→Mg	$\nabla^2\rho$-O5→Mg
QE-LDA	NF	NF	0.04080	0.23085	0.03962	0.21617
QE-PBE	NF	NF	0.03789	0.28127	0.03681	0.22526
QE-PW91	0.04018	0.35523	0.03787	0.28149	0.03678	0.22518
VASP-LDA	0.04327	0.29681	0.04080	0.23085	0.03962	0.21617
VASP-PBE	0.04203	0.30854	0.03960	0.23747	0.03852	0.17335
VASP-PW91	0.04207	0.30843	0.03963	0.23722	0.03854	0.17311

S6. Energy Convergence with computational conditions

Several energy convergence tests were performed on the urea crystal as a compromise between crystal complexity and computational cost. We take as baseline the production settings used in the main text (600 eV plane-wave energy cutoff, 1×10^{-6} eV SCF energy threshold and a $8 \times 8 \times 10$ k-points grid, and test the energy convergence of these parameters both in QE and VASP with the LDA DFA. The QE results (in Rydberg) are shown in Table S7 and the VASP ones (in eV) in Table S8, respectively. Provided that the the SCF threshold has no influence on the energy, this parameter was only tested in QE. The size of the hard and smooth FFT grids was also tested. Increasing their sizes above those predefined by the PREC=Accurate Keyword in VASP has no effect on the total energy (variations smaller than 10^{-5} eV) but has a profound effect on the density, as evidenced in the next sections. Please note that all data in this and the following sections are LDA results. Similar results are found with other functional approximations.

Table S7. *QE Energy convergence in the urea crystal upon variation of the cutoff energy (E_c), the SCF threshold (E_t) and the size of the k-point mesh (n), all taken from the baseline described above.*

E_c (eV)	E (Ry)	m, ($E_t = 10^{-m}$ eV)	E (Ry)	n	E (Ry)
400	-235.98075891	4	-236.37381033	2	-236.37137247
500	-236.33312754	5	-236.37381235	4	-236.37383606
600	-236.37381273	6	-236.37381273	6	-236.37381157
700	-236.37688581	7	-236.37381276	8	-236.37381516
800	-236.37933885	8	-236.37381276	10	-236.37381766

Table S8. *VASP Energy convergence in the urea crystal upon variation of the cutoff energy (E_c) and the size of the k-point mesh (n), all taken from the baseline described above.*

E_c (eV)	E (eV)	n	E (eV)
400	-104.81610409	2	-104.81419278
500	-104.81070054	4	-104.83957508
600	-104.83915716	6	-104.83911917
700	-104.87774631	8	-104.83916050
800	-104.89749616	10	-104.83916535

S7. Density convergence with computational conditions

We have tested the evolution of ρ both in QE and VASP on fully energy-converged urea calculations that use an energy cutoff of 800 eV, a $10 \times 10 \times 10$ k-point grid and SCF threshold of 10^{-6} eV. We have used fine $n \times n \times n$ FFT grids with $n = 80, 100, 120, 140, 180$ points. Since the density along the CO line has been found to be most sensitive to computational conditions, Fig. S4 shows that even in these conditions VASP and QE converge to clearly different results.

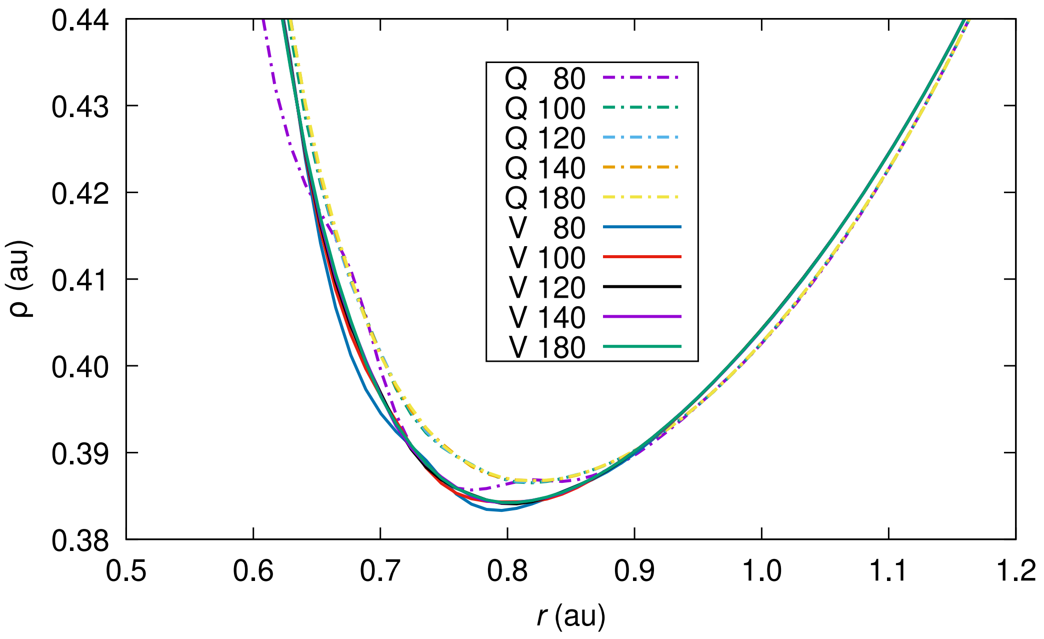

Fig. S4. Density along the CO line in urea (r taken from the C atom) as the size of the FFT grid is varied from a fully energy-converged calculation. Q stands for QE and V for VASP calculations.

S8. Critical point convergence

Taking the baseline calculation described in the main text, Tables S9 and S10 contain the density and Laplacian at the CO and CN critical points for urea as the cutoff energy is changed for QE and VASP, respectively. Some CPs were not found in QE calculations.

Table S9. *QE critical points in the urea crystal upon variation of the cutoff energy (E_c), all taken from the baseline described above.*

E_c (eV)	ρ_{bcp}(CO) (au)	$\nabla^2\rho_{bcp}$(CO) (au)	ρ_{bcp}(CN) (au)	$\nabla^2\rho_{bcp}$(CN)
400	3.76323640E-01	8.11309999E-01	3.43787631E-01	-1.04841320E+00
500			3.46588695E-01	-1.11587219E+00
600	3.80563931E-01	2.54075736E+00	3.47621702E-01	-1.14026633E+00
700	3.88277180E-01	3.58876739E-01	3.47774577E-01	-1.18334855E+00
800			3.47803677E-01	-1.17221050E+00

Table S10. *VASP critical points in the urea crystal upon variation of the cutoff energy (E_c), all taken from the baseline described above.*

E_c (eV)	ρ_{bcp}(CO) (au)	$\nabla^2\rho_{bcp}$(CO) (au)	ρ_{bcp}(CN) (au)	$\nabla^2\rho_{bcp}$(CN)
400	3.79418514E-01	1.50113312E+00	3.44200806E-01	-9.67731165E-01
500	3.81984826E-01	8.14208695E-01	3.45333322E-01	-9.66837503E-01
600	3.82101498E-01	7.06276407E-01	3.45907545E-01	-9.60252198E-01
700	3.82649019E-01	7.13784197E-01	3.46705585E-01	-1.01223585E+00
800	3.83921003E-01	4.81372187E-01	3.47444014E-01	-1.13519257E+00

Convergence was also checked in VASP at a cutoff energy of 800 eV in Table S13. As it can be seen, densities at CPs coverge rather quickly with the FFT grid size, but Laplacians do not.

Table S11. *VASP critical points in the urea crystal upon variation of the FFT grid size, all taken from the baseline described above.*

FFT size	ρ_{bcp}(CO) (au)	$\nabla^2\rho_{bcp}$(CO) (au)	ρ_{bcp}(CN) (au)	$\nabla^2\rho_{bcp}$(CN)
80	3.83316833E-01	6.65469533E-01	3.47406313E-01	-1.07415813E+00
100			3.47428862E-01	-1.09210957E+00
120	3.84063413E-01	2.78496924E-01	3.47435720E-01	-1.08315924E+00
140	3.84199604E-01	-2.86018387E-01	3.47443538E-01	-1.10695199E+00
180	3.84201558E-01	-3.13108274E-02	3.47447391E-01	-1.11777364E+00

S9. Sensitivity to change in the position of CPs

It is well known that the position of the bond critical point in polar bonds like that in CO is rather sensitive to basis sets and other computational conditions. It is thus relevant to check whether densities or Laplacians do not converge easily because of this position dependency but otherwise are stable at the same spatial position. We have thus taken the position of the BCPs in urea in the converged calculation reported in the main text for the VASP code (crystallographic positions $(0.00000000, 0.50000000, 0.41837714)$ and $(0.94746956, 0.44746956, 0.27391939)$ for the CO and CN BCPs, respectively, and test the density and Laplacian as we change the

FFT grids at a cutoff energy of 800 eV.

Table S12. *QE critical points in the urea crystal upon variation of FFT grid size, all taken from the baseline described above.*

FFT	ρ_{bcp}(CO) (au)	$\nabla^2\rho_{bcp}$(CO) (au)	ρ_{bcp}(CN) (au)	$\nabla^2\rho_{bcp}$(CN)
80	3.860931193E-01	1.948646735E-01	3.482074878E-01	-1.134027989E+00
100	3.868506861E-01	2.645249896E-01	3.482028329E-01	-1.114245392E+00
120	3.869065809E-01	1.757024362E-01	3.482081191E-01	-1.111808602E+00
140	3.870007749E-01	-4.825238283E-01	3.482055484E-01	-1.111182555E+00
180	3.870297868E-01	-1.319781731E-01	3.481980106E-01	-1.102779987E+00

Table S13. *VASP critical points in the urea crystal upon variation of FFT grid size, all taken from the baseline described above.*

FFT	ρ_{bcp}(CO) (au)	$\nabla^2\rho_{bcp}$(CO) (au)	ρ_{bcp}(CN) (au)	$\nabla^2\rho_{bcp}$(CN)
80	3.833537621E-01	3.645770427E-01	3.474306926E-01	-1.048332621E+00
100	3.843226198E-01	-1.744866939E+00	3.474488703E-01	-1.064946062E+00
120	3.840871465E-01	4.497610156E-01	3.474569255E-01	-1.056698100E+00
140	3.841997373E-01	-3.073219079E-01	3.474636773E-01	-1.079220574E+00
180	3.842090220E-01	8.666269631E-02	3.474680611E-01	-1.089534904E+00

All in all, it seems clear that Laplacians along the CO line cannot be fully trusted, and that even isolating the CP position problem, QE and VASP densities seem to converge, once again, to different points.
